# What Matters in Help-Seeking and Disclosure Intent of Intimate Partner Violence During the COVID-19 Pandemic: Similarities and Differences Across Demographic Groups

**DOI:** 10.3390/ijerph23030319

**Published:** 2026-03-04

**Authors:** Christina Palantza, Maxine Davis, Anke B. Witteveen, Diana Padilla Medina

**Affiliations:** 1 Department of Population Health Sciences, Bristol Medical School, University of Bristol, Bristol BS8 2PN, UK; 2 Rutgers School of Social Work, Rutgers University, New Brunswick, NJ 08901, USA; maxine.davis@rutgers.edu; 3 Department of Clinical, Neuro- & Developmental Psychology, Vrije Universiteit Amsterdam, 1081 BT Amsterdam, The Netherlands; a.b.witteveen@vu.nl; 4 Graduate School of Social Work Beatriz Lassalle, University of Puerto Rico, San Juan, PR 00917, USA; diana.padilla@upr.edu

**Keywords:** Intimate Partner Violence (IPV), COVID-19, help-seeking, disclosure

## Abstract

**Highlights:**

**Public health relevance—How does this work relate to a public health issue?**

- Intimate Partner Violence (IPV) is a pressing public health issue internationally
- The COVID-19 pandemic was a severe public health crisis that exacerbated IPV

**Public health significance—Why is this work of significance to public health?**

- Learnings from the COVID-19 pandemic are useful to inform how to tackle IPV in future crises
- Key demographic differences in help-seeking for IPV are examined

**Public health implications—What are the key implications or messages for practitioners, policy makers and/or researchers in public health?**

- IPV screening should be prioritized, especially in mental health services
- Programs and services directed to vulnerable groups should be protected in emergency situations

**Abstract:**

The COVID-19 pandemic increased Intimate Partner Violence (IPV) internationally and disrupted health services. The pandemic also exacerbated risk factors linked to IPV, such as deteriorating mental health. As access to health care became restricted, IPV survivors faced barriers to help-seeking. No study has examined the factors related to IPV help-seeking intent during the pandemic, which might differ from actual behavior. The aim is to examine the impact of number of COVID-19 cases and health on IPV help-seeking and disclosure intent. A cross-sectional survey in the USA in April 2020 assessed health status, IPV (victimization and perpetration), help-seeking and disclosure intent. Linear models were used (N = 1346). Upper income positively correlated with help-seeking and disclosure intent. In terms of number of COVID-19 cases and PTSD symptomology with help-seeking intent, changes in daily life correlated positively with disclosure intent, but experience of violence correlated negatively. There were significant demographic differences. Inconsistency in the reporting of violence across scales was a notable issue. The findings on mental health support the existing literature. Healthcare providers in all settings should prioritize IPV screening. Access to care should be maximized through continued improvement/expansion of online services and policy changes that remove barriers (such as lapse in insurance coverage or financial burden).

## 1. Introduction

Intimate Partner Violence (IPV) is any form of aggression or violence towards one’s intimate partner [1] and a major public health issue across the globe [2]. The COVID-19 pandemic, which started affecting a great deal of the world in March 2020, increased the prevalence, severity and frequency of IPV internationally [3]. Help-seeking for IPV was also affected by the context of the pandemic, with decreases observed in the early stages, but sharp increases in the later stages [4]. Access to IPV services worsened during the pandemic [5,6] as it would be expected, given the lockdowns or social distancing measures, and the overloading of health systems. At the same time, health and social care delivery became more challenging [7,8]. This pandemic combined different adverse circumstances, making it a particularly challenging rare event. It offers many uniquely valuable lessons to inform potential future crises, either pandemics or other emergency situations, such as natural disasters, mass displacement, and financial crises, all of which are more likely given the progression of climate change [9]. Such crises share some mechanisms with the COVID-19 pandemic that affect IPV rates, help-seeking and service provision [10,11]. Therefore, it is imperative to make the most of all the data that were collected during the COVID-19 pandemic, especially about IPV, to inform public health policy and organization of services for such potential future crises, as well as general system resilience and sustainability [12].

The demand for services was high but not always met [7,13] with IPV worsening and barriers to help-seeking rising [14]. Discrepancies have been found in the reports of IPV survivors and providers about the changes incurred by the pandemic [15]. There is some evidence that help-seeking did not actually decrease during the pandemic, but rather referrals decreased due to practical constraints [16]. When screened by healthcare providers disclosures were low compared to the actual prevalence [17]. It is acknowledged that there has been a significant gap between the need and access to services [15], especially for survivors belonging to previously disadvantaged and vulnerable groups [18]. This means that many IPV survivors, probably more than usual, intended to seek help but did not manage to. It is generally accepted that intent does not always lead to behavior [19,20] and that in emergency situations, such as the pandemic, there are rapid changes in how many manage to act on their intentions [21]. Indeed, an American study showed that 22% of IPV survivors were interested in seeking help, but did not manage to, and two out of the three main reasons cited were fear of being infected with the coronavirus and the closure or limited capacity of many services [22]. Knowing which IPV survivors intend to seek help could be useful in order to have a more realistic picture of the actual need for services in emergency contexts. It would also help identify more concrete and targeted strategies to actively support those that face additional difficulties due to the situation itself and the structural inequalities it might exacerbate [21,23].

Formal help-seeking is necessarily preceded by disclosure of IPV to professionals. The most common barriers to disclosure are fear of retaliation by the partner [24,25,26] and feeling trapped or dependent on the partner [27]. For many, the stay-at-home and shelter-in-place orders created an environment which increased such feelings. On top of that, research spanning decades indicates that IPV-affected individuals are much more comfortable and likely to disclose IPV when there is a personal, compassionate and understanding approach by the provider [28]. Such an approach is less likely to be adopted by health-care professionals in the context of widespread disaster (i.e., the pandemic), while providers face numerous challenges/uncertainty in the workplace [8], experience increased private life stressors, and heavier workloads. Moreover, survivors tend not to communicate with providers about IPV when they believe there is a lack of time [29], which was a reality throughout the pandemic—especially during the earliest stage, and studies have shown that there were missed opportunities for IPV screening [30]. Survivors are more likely to disclose IPV when screened for it, rather than to mention it themselves to the providers voluntarily without any probe [31,32]. However, an encouraging and supportive approach is necessary for the screening to be acceptable [33]. An analysis of data from 33 countries during the pandemic showed that only 12% of general practitioners screened more for IPV; only 11% had more IPV disclosures and they generally did not communicate proactively about IPV [34].

The COVID-19 pandemic has had widespread effects on other areas of public health as well, with mental health emerging as a particularly vulnerable one [35]. Poor mental health and substance abuse are among the most common and strongest correlates of IPV. IPV has been shown to take a heavy toll on survivors’ mental health [36,37], but perpetrators very often suffer from mental disorders and abuse substances too [38,39]. Substance abuse, especially of alcohol, increased in the context of the pandemic [40], and this has been directly connected to increased IPV risk [41,42]. Mental health issues play a significant role in help-seeking behaviors as well [43], with some studies identifying mental health issues as a barrier to help-seeking [44], while other studies describe experiencing poor mental health as motivation to seek help [45,46]. This is corroborated in a North American survey from the pandemic that identified poor mental health as a barrier to help-seeking for IPV [47]. Apart from general mental distress, post-traumatic stress symptoms are one of the most common mental health consequences of IPV [48], and they have been linked with increased help-seeking [49]. It is also common among IPV perpetrators [50].

In addition to the empirical studies, there are several theoretical models about help-seeking for IPV that acknowledge the significant role of mental health in seeking help, even in conflicting ways. The IPV stigmatization model [51] implies that poor mental health is a barrier, particularly as a correlate of stigma, which is often intertwined with PTSD [52] and depression symptomatology [53]. The most widely used theoretical framework of help-seeking for IPV is that of Liang and colleagues [54]. This framework proposes three stages to IPV help-seeking: 1. Definition of the problem; 2. Decision to seek help; 3. Selection of a source of support. The authors acknowledge intrapersonal and interpersonal layers in all of these stages. Poor mental health is an internal factor which might hamper the definition of IPV as a problem when guilt as a mental distress symptom is intense, or it might motivate help-seeking when the survivor realizes the lack of prospects for improvement without help. At the final stage, mental health professionals can be considered and/or selected as a source of support. Other models are often used in the IPV field, such as Andersen’s [55] and Schreiber, Renneberg, & Maercker’s [56], even though these models were originally developed for general healthcare use or general interpersonal violence. Andersen’s model comprises three types of factors: 1. Predisposing ones, which create the requirement for healthcare, 2. Enabling ones, i.e., external objective aspects, such as availability of services and income of the individual, 3. Need-related variables, both as evaluated and perceived need. Mental health issues could serve as a predisposing or need-related factor, thus enabling help-seeking, as an individual feels distressed or they do not function as they would wish, so healthcare is required to ease such feelings. Applying Schreiber and colleagues’ model—which focuses on psychosocial care seeking after traumatization—PTSD symptoms, such as post-traumatic avoidance, guilt and shame, are theorized to negatively influence the perception of IPV as a problem and the wish for treatment. These stages precede the treatment intention, the step prior to help-seeking, which is deemed to be influenced mostly by external and practical aspects. It is also clear that help-seeking intent is placed at the middle-level stage in Liang’s and Andersen’s models, where both internal and external factors are at play. The Andersen model has been tested along with the Theory of Planned Behavior (TPB) [57], and it has been suggested that it is more valid to integrate them, as they focus on different parts of the same process [49]. The integrated model proposed [49] highlights the key roles of post-traumatic stress symptoms, the perceived effectiveness of the resources and lower perceived controllability of IPV. The latter two were likely affected by the pandemic, as health services were perceived as overloaded and some IPV survivors might have thought that confinement makes the violence or the control worse, and could thus ease as soon as things returned to normal.

Improving mental health may be seen as second to creating a need for services when compared to the violence itself [58]; thus, they can serve as the predisposing factors constituting the need. Severity of violence and experiencing multiple types of violence (psychological, physical, sexual) at once have been consistently shown to lead to help-seeking [59,60,61,62,63]. Therefore, different types of violence need to be considered when assessing factors influencing help-seeking.

Apart from mental health and violence, there is an instrumental role of structural and practical factors related to help-seeking, the external factors acknowledged by the theoretical models. During the COVID-19 pandemic, these systemic barriers—such as restricted mobility, service availability, and financial instability—may have had a greater impact than interpersonal factors. Limited service availability and restricted mobility were usually more severe in areas with higher numbers of COVID-19 cases. In addition to the sheer numbers of cases, the pandemic was a major stressful life event that brought about significant changes in everyone’s lives, especially IPV survivors, and their capacity to seek help [64]. Evaluating the relative influence of number of COVID-19 cases and changes in daily life, alongside mental health and violence severity, is essential for a comprehensive understanding of IPV-related help-seeking.

In addition to intrapersonal and external factors, the public’s top concern during the COVID-19 pandemic was physical health. Physical health is well known to be particularly compromised among survivors of IPV [61,65]. People with severe or chronic physical health issues would presumably prioritize these conditions instead of IPV during the pandemic for several reasons. This was evident in another North American survey, which also found mediating effects of poor physical health between IPV and barriers to formal help-seeking [47]. For example, being reluctant to visit services out of fear of getting infected with COVID-19, or avoiding leaving the house, even when allowed by social distancing measures, which was confirmed in the study by Shyrokonis et al. [22]. With that in mind, we hypothesized that poor physical health was associated with decreased help-seeking for IPV during the pandemic.

To more efficiently manage the rising need for services in their limited availability, in the context of the COVID-19 pandemic crisis, it would have been important to pinpoint which factors impacted help-seeking and disclosure [66], but correlates to such changes have not been investigate to their full extent [67]. Help-seeking in the pandemic has been researched mostly through the records of health services, the police, and helplines [10,16,68,69,70], and surveys have been conducted in smaller regions [22], or in perinatal populations [71]. In order to capture the full scope of help-seeking need in the pandemic and the factors that influence it, it is necessary to study the intent to seek help and the intent to disclose IPV among individuals affected by IPV in the general population, and not just service records. The Canadian survey by Idriss-Wheeler and colleagues [47] did find increased barriers to help-seeking among IPV survivors compared to the general population during the pandemic which were correlated with low income, caregiving commitments, perceived community violence, and poor physical and mental health. However, the Canadian health system is vastly different from that of the USA, so it is likely that the availability and accessibility of services are not comparable. Moreover, this study focused on individuals that self-identified as IPV survivors, while it is well-known that victimized individuals do not always realize that what they are experiencing qualifies as abuse [72]. Lastly, the study focused on perceived mental and physical health. Therefore, it is important to examine if these findings still hold in a different health system, also with more sensitive IPV detection and more objective measures of mental and physical health.

Another important aspect that has not been investigated comprehensively is the specific evidence for different demographic groups. It is well acknowledged that there are considerable structural inequalities and vulnerability characteristics that elevate IPV risk and hamper help-seeking in crises [4,64]. It is also established that IPV, help-seeking and mental health are experienced very differently across genders [72], ethnicities [4,73], age groups [74] and income levels [75]. Therefore, it is worth examining the correlates separately for each group, since they are conceptually discrete groups that do not share the same level of variance in all their characteristics [76], and interpretation is more straightforward for each subgroup [77].

This objective for greater understanding is pressing, as climate change is speculated to incur more crises, including pandemics [78], which typically cause spikes in IPV and hamper help-seeking, especially among those with pre-existing vulnerability [79,80]. To provide this more nuanced insight into the demographic differences in the intent to disclose IPV and seek help (which stipulates the actual need for services), the primary research question of this study is: What is the association of the number of COVID-19 cases in the state, mental and physical health, alcohol abuse, PTSD and type of IPV with help-seeking and disclosure intent and what are the demographic differences? In order to investigate potential demographic differences in further detail, the following research sub-questions were examined, as presented in Table 1

## 2. Materials and Methods

### 2.1. Design, Setting and Ethics

This study is a secondary analysis of a subset of the sample of a cross-sectional survey which was carried out online among the general population in the United States from 15 April to 1 May 2020, using the Qualtrics platform. The participants gave written informed consent through Qualtrics. The original survey was ethically approved by the Institutional Review Board (IRB) of the University of Texas at Arlington (Protocol number 2020-0193).

### 2.2. Participants

The convenience sample was an internet panel of individuals who have signed up to the Qualtrics platform for any survey, driven primarily by the financial rewards of the surveys [81]. The eligibility criteria were living in the USA and being 18 years old or more. It is estimated generally that the people expressing interest in a survey are a tenth of those that see it in their Qualtrics dashboard of surveys they might be eligible for, but Qualtrics does not track these data for any survey [81]. Quota sampling by gender, age and ethnicity was used to achieve a balanced and representative sample of the US general population [81]. Recruitment for survey participation was described in generic terms to decrease the chance that IPV-involved individuals would increasingly approach or avoid the survey [81]. IPV prevalence data in the entire sample was consistent with prior nationally collected data in the U.S. Only participants that had perpetrated IPV or were victimized by IPV were included in the present study, because the outcomes of help-seeking and disclosure intent for IPV are not relevant to people without any experience of IPV. That meant answering positively to at least one question on psychological, physical, or sexual victimization and/or perpetration in any of the IPV scales.

### 2.3. Data Collection and Management

All participants completed the questionnaires online. The response rate was ~6%, which is typical for internet panels [82]. Responses that were duplicates, suspected bots, completed suspiciously fast (under 479 s), missing most of the questions, containing sensitive data, or violating the quota were removed (Ν = 1705 out of 3750, with n = 400 being removed after running a test for quality). The data were anonymized. A data sharing agreement was signed between the first authors of the parent study and the present study, and the data were transferred and stored securely through a General Data Protection Regulation-compliant platform. The data for this analysis were first accessed on 2 March 2022.

### 2.4. Operationalization of Variables

#### 2.4.1. Independent Variables

The independent variables were physical health, mental health, alcohol abuse, post-traumatic stress disorder (PTSD) symptoms, number of COVID-19 cases in the state, changes in daily life due to the pandemic, psychological victimization, perpetration of psychological violence, physical victimization, perpetration of physical violence, sexual victimization, and perpetration of sexual violence.

The classification of physical health status from the American Society of Anesthesiologists [83] was adapted to classify health status. The questionnaire included a list of several physical conditions and health symptoms. The participants could answer if they had them currently, in the past, or never. The final classification was: “1: healthy” = having fewer than five of the symptoms listed, “2: mild disease” = having a mild disease, such as an allergy or more than five of the symptoms listed, “3: severe yet not incapacitating disease” = having a serious disease that is not constantly life threatening or incapacitating, such as an autoimmune disease, or diabetes, “4: current constantly life threatening disease” = cancer, and “5: history of serious disease” = checking off any serious disease but only in the past.

Overall mental health was assessed with the shortened version of the Brief Symptom Inventory (BSI-18) [84]. Each item is scored 0–3, so the range of scores is 0–72. It has been found to have adequate psychometric properties [84]. The reliability in this sample was excellent with a Cronbach’s alpha of 0.943. Since PTSD is one of the most common disorders among people involved in IPV [36], it was included as a separate variable and measured with the International Trauma Questionnaire (ITQ) [85,86]. It consists of six items, representing the three clusters of PTSD symptoms (reexperiencing, avoidance and sense of threat). The items are scored 0–4, and the total score is used. It has high reliability in the US population [85], and it was also highly reliable in this sample with a Cronbach’s alpha of 0.958. The survey included questions on mental disorder diagnoses, in the same fashion as the physical ones, and a variable indicating having any mental diagnosis was also used, because individuals who have received a diagnosis often face increased barriers in accessing healthcare services [87,88]. Alcohol abuse was one of the conditions listed, so the participants self-reported whether they had it currently, in the past or never.

The number of COVID-19 cases in the state was operationalized as the number of cases in the state on 1 May 2020, in the same manner as in Davis and colleagues [81] [i.e., an ordinal variable of four levels (0–4999 cases/5000–9999/10,000–23,999/24,000+)].

Subjective change in one’s daily life might implicitly affect their mental health and decisions regarding help-seeking, so an ordinal variable on changes in daily life was included, based on dichotomous questions about changes experienced. The levels were: 0 = no changes, 1 = small changes, such changes in social life and starting to work from home, 2 = severe changes, such as having lost one’s job or changes in family life.

Perpetration and victimization of IPV were assessed with three different scales, as the survey’s original purpose was to assess the psychometric properties of a newly developed IPV tool. The three scales were the 5-item Extended-Hurt/Insult/Threaten/Scream (Modified E-HITS) [89], the Jellinek inventory for assessing partner violence (J-IPV) [90], and the Revised Conflict Tactics Scale (CTS-2) [91]. All of these tools have shown satisfactory psychometric properties; the E-HITS has been identified as an accurate and acceptable screener [89]; the J-IPV is a very low-burden tool, as it consists only of 4 items and it is a very robust screener [92]. The CTS-2 is more elaborate and serves as a reference. Nevertheless, the participants of the present study often gave inconsistent answers across IPV scales in the three measures. This has been attributed to gender and age disparities in phrasing [93], but assuming that some participants could be ambivalent about reporting, a positive answer in any scale was deemed as sufficient. Further work on the reporting inconsistencies is under preparation. All these scales include questions both on victimization and perpetration. The weighted kappa value for the items of psychological perpetration across scales was concerningly low (ranging from 0.273 to 0.486), which also held for psychological victimization ranging from 0.038 to 0.372. For physical perpetration weighted Cohen’s kappa ranged from 0.341 to 0.524 and for victimization from 0.330 to 0.514. For sexual perpetration the weighted Cohen’s kappa between E-HITS and CTS was also too low at 0.501, and for victimization at 0.538.

Psychological violence was coded as a continuous variable, because psychological violence has been shown to have a variety of nuances with different impacts on the survivors [94]. The coding was done in scores: 0 = no violence, 1 = only yelling, 2 = only insults or only threats, 3 = insults and yelling, or threats and yelling, or insults and threats. Past psychological victimization and perpetration were separate variables, as history of psychological abuse can have a lasting effect on mental health [95]. Physical and sexual violence were both coded dichotomously, either in absence or presence of any violence.

#### 2.4.2. Outcome Variables

Help-seeking intent was measured with one item, “If a healthcare provider, such as your primary care provider, were to ask you such questions, to what extent would you be likely to be interested in hearing about resources that may be of help?”, answered with a Likert-type scale from “extremely unlikely” to “extremely likely”. Disclosure intent was measured with two items, phrased as: “If a healthcare provider, such as your primary care provider, were to ask you such questions, to what extent would you be likely to discuss these experiences with the provider?”, and “If a healthcare provider, such as your primary care provider, were to ask you such questions, to what extent would you be likely to answer the questions honestly?”. These questions were phrased this way because when the survey was designed, prior to the outbreak of COVID-19, they were intended to explore the appropriateness of the phrasing for IPV screening. The answering options were the same as help-seeking intent, and the final score was the average of two items. The outcome questions used corresponded to the E-HITS.

### 2.5. Statistical Analysis

Descriptive statistics of all the variables and Pearson and Spearman correlations between every pair of variables were calculated in SPSS 28 [96]. Linear regression models were chosen over mixed effects linear regression because even though the data showed some clustering by state, the Intra-cluster Correlation Coefficient (ICC) was <0.05 and clustering did not improve model fit. The model was run separately for victimization and perpetration, because the items answered by participants screening positive for perpetration or victimization were coded separately. The model was also run separately for each type of violence to examine more carefully if there are distinct associations for different types of violence. The mental-health-related variables (BSI score, PTSD score, any mental disorder diagnosis, alcohol abuse) were highly correlated, but the collinearity diagnostics of SPSS showed no variable having a variance inflation factor < 3. There were some high condition indices, a few at 15–16 and one at 29, but the variance proportions did not reach 0.70 for more than one variable in the corresponding tests. Many participants had missing data in at least one variable, so in order to retain statistical power, the missing data were imputed with chained equations using the “mice” R package, and the regression results were pooled according to Rubin’s rules. Two sensitivity analyses were conducted as well, one considering only the participants who reported violence in the scale of interest for each type of violence (J-IPV for physical violence, and E-HITS for all other types of violence), and one considering only the participants who reported violence on these scales, and their answers were consistent across all scales. The assumptions of linear models were met.

The literature stresses important differences in help-seeking barriers among genders [97,98] and people with low income [99], in disclosure among sexual minorities [100] and older adults [101]. Hence, Kruskal–Wallis tests were run for the independent and dependent variables by gender, age, income level, and ethnicity, as well as corresponding subgroup analyses of the models, because the outcomes followed a gamma distribution, skewed towards higher values. We opted for subgroup analyses instead of moderation analysis because the variables are discrete categories with conceptual significance, and interpretability is facilitated. It is worth knowing the correlates for all demographic groups, even if there is not a statistically significant interaction.

## 3. Results

### 3.1. Sample Descriptives

After excluding those who were not IPV survivors or perpetrators in the past six months, the final sample consisted of 1346 participants. The total sample of the original study was N = 2045, which means that the prevalence of any IPV of any type and severity in the original sample was 65.82%. This high prevalence is most likely due to the combination of two factors: first and foremost, the data were collected in the earliest stage of the pandemic, when the stress in families was very high; a systematic review focusing on IPV in the USA during the pandemic highlights and exponential increase in IPV due to pandemic-related stressors [102]; secondly the use of the CTS may have inflated the prevalence of IPV in this general population sample (which also included younger individuals) where the context and the impact of the acts were not taken into account [103,104]. The final sample of this study was balanced in terms of gender, with 47.3% females, and 2.5% people had a different gender identity or their identity was not represented (detailed descriptives in Table 2). Emerging adults (young people of ages 18–29) were a fourth of the sample, adults more than a third (35.3%), and older adults 12.8%. Nearly 80% of the sample was heterosexual, and almost 10% had an orientation other than hetero, homo, or bisexual (detailed descriptives in Table 2). Regarding ethnicity, 12.9% was African American, and 13.9% Latinx participants or Caribbean, while 6.5% identified as multi-ethnic. Almost three-fourths were employed (73.3%), more than a third had a middle annual income, and a third upper-middle income. Three-quarters were Christian (44.4% Catholic, 23.1% Protestant, and 10.4% other Christian). More than 90% were born in the USA. The states with the most participants were New York (14.3%) and California (12.3%). Nearly half had faced mild changes in their daily life because of COVID-19, and one quarter had been diagnosed with COVID-19 or someone in their close environment had been infected. A little more than 40% had a mental disorder diagnosis in their lifetime, and less than a third were classified as healthy. Approximately 90% identified both as a survivor and a perpetrator. This is probably due to the use of CTS which is known to show an inflated gender symmetry, because of the lack of context that overlooks the dynamics, for example not differentiating acts of self-defense [105,106]. Fourteen percent had lifetime alcohol abuse. Past six months physical perpetration and victimization were between 40 and 44% respectively, and sexual perpetration and victimization between 31 and 34% respectively. More details can be found in Table 2. Even though quota sampling was used and the sample is diverse, it is not representative of the USA population [81]. In the following sections with the results of the regression models testing correlates of the outcomes, we only mention the correlates that were confirmed in the majority of the models. The full set of variables that reached significance is presented in the tables.

### 3.2. Help-Seeking Intent

#### 3.2.1. RQ 1.1: What Predicted Intention to Seek Help for IPV?

Number of COVID-19 cases in the state, upper-middle or high income and PTSD score significantly predicted a higher help-seeking intent in all analyses. Severe changes in daily life (e.g., having lost their job or changes in family life) were positively correlated with help-seeking intent among victims only in the main analysis. Being an adult (30–44) perpetrator or a Native American victim were also positively correlated with help-seeking intent in the main analysis, but not the sensitivity analysis, with consistent reporting of violence. Being a female victim was negatively correlated in the main analysis, while in the sensitivity analysis being a female perpetrator was negatively correlated with help-seeking intent. A finding that was yielded only from the sensitivity analyses was negative correlation of psychological and physical perpetration and sexual victimization with help-seeking intent. The results of the main analysis are presented in Table 3.

#### 3.2.2. RQ 1.2: Did Correlates of Help-Seeking Intention Differ by Gender?

**Figure 1:**
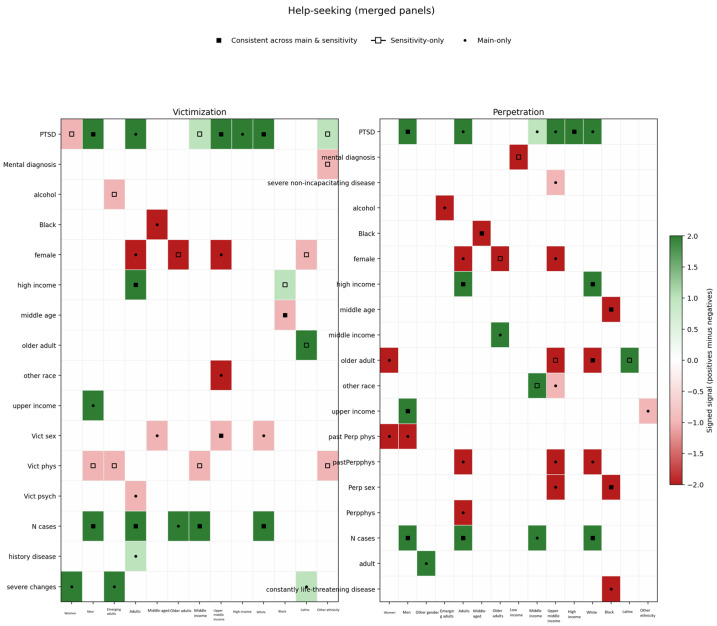
Robust correlates of help-seeking intent by demographic group.

Kruskal–Wallis tests showed different help-seeking intent among male survivors and among perpetrators identifying as “other”, but only in the main analysis. In the sensitivity analysis of consistent answers across IPV scales, female perpetrators showed a lower help-seeking intent. The subgroup analysis among males gave the exact same results as the main analysis, with PTSD, number of COVID-19 cases in the state and upper income positively correlated with help-seeking. For females there were discrepancies between the main and the sensitivity analyses; the most robust correlate was severe changes in daily life, that positively correlated with help-seeking intent among victims only in the main analysis. The participants identifying as other gender were few and most models could not converge, which means that these results cannot be interpreted. The results on gender differences are presented in Figure 1.

#### 3.2.3. RQ 1.3: Did Correlates of Help-Seeking Intention Differ by Age?

Regarding age groups, adults had significantly higher intent to seek help than emerging adults and middle aged in the main analysis, but in the sensitivity analysis the only difference was that the middle-aged perpetrators had a lower intent than adults. The different analyses of emerging adults gave different results; the most consistent correlate was severe changes in daily life among victims in the main analysis, and some negative correlations of alcohol abuse. Among adults, the results were quite similar to the main analysis, with upper income, PTSD and number of cases in the state positive correlates, and in the main analysis being a female victim was negatively correlated with help-seeking intent. Among the middle-aged, barely any variable reached significance in the sensitivity analyses, and in the main analysis being Black was a consistent negative correlate. There were discrepancies between the main and sensitivity analyses for older adult participants as well. In the main analysis, number of cases in the state correlated with higher help-seeking intent among victims, while among perpetrators middle income was a positive correlate. In the sensitivity analysis of consistent answers female gender was negatively correlated with help-seeking both among victims and perpetrators. The results on age differences are presented in Figure 1.

#### 3.2.4. RQ 1.4: Did Correlates of Help-Seeking Intention Differ by Income Level?

Kruskal–Wallis tests detected that the two lower income groups were different from the two higher groups. No correlates were consistently significant among those with low income. Among those with a middle income, number of cases was a significant correlate in most analyses, even though not fully confirmed in the sensitivity analysis. In the upper-middle-income group, PTSD was positively associated, female gender and victim of “other” ethnicity negatively associated, throughout the main analysis, but hardly replicated in the sensitivity analysis. For high-income participants, PTSD was a consistent positive correlate but barely replicated in the sensitivity analysis. The results on income differences are presented in Figure 1.

#### 3.2.5. RQ 1.5: Did Correlates of Help-Seeking Intent Differ by Ethnicity?

There were hardly any differences across ethnicities in help-seeking intent. Regarding correlates, the results of the White participants group were very close to the full analysis, with number of cases, high income (albeit only among perpetrators) and PTSD being the most robust correlates, replicated in the sensitivity analyses. Among Black participants being middle aged was a consistent negative correlate. Among the Latinx participants, barely any variable was significant in the main analysis, but in the sensitivity analysis of consistent answers older age was positively correlated. Among participants of other ethnicities, no variable emerged across multiple models. The results on ethnicity differences are presented in Figure 1.

### 3.3. Disclosure Intent

#### 3.3.1. RQ 2.1: What Predicted Intention to Disclose IPV?

Violence of most types, both victimization and perpetration, was consistently negatively correlated with disclosure intent. Severe changes in daily life and high income were positively correlated with disclosure intent. These were confirmed in the sensitivity analyses. BSI score was also negatively correlated, except when tested along with psychological violence, and PTSD was positively correlated, but these were not confirmed in the sensitivity analyses. Being Black was negatively correlated only in the main analysis and older age was positively correlated only in the sensitivity analyses of consistent answers. The results of the main analysis are presented in Table 4.

#### 3.3.2. RQ 2.2: Did Correlates of Intention to Disclose IPV Differ by Gender?

**Figure 2:**
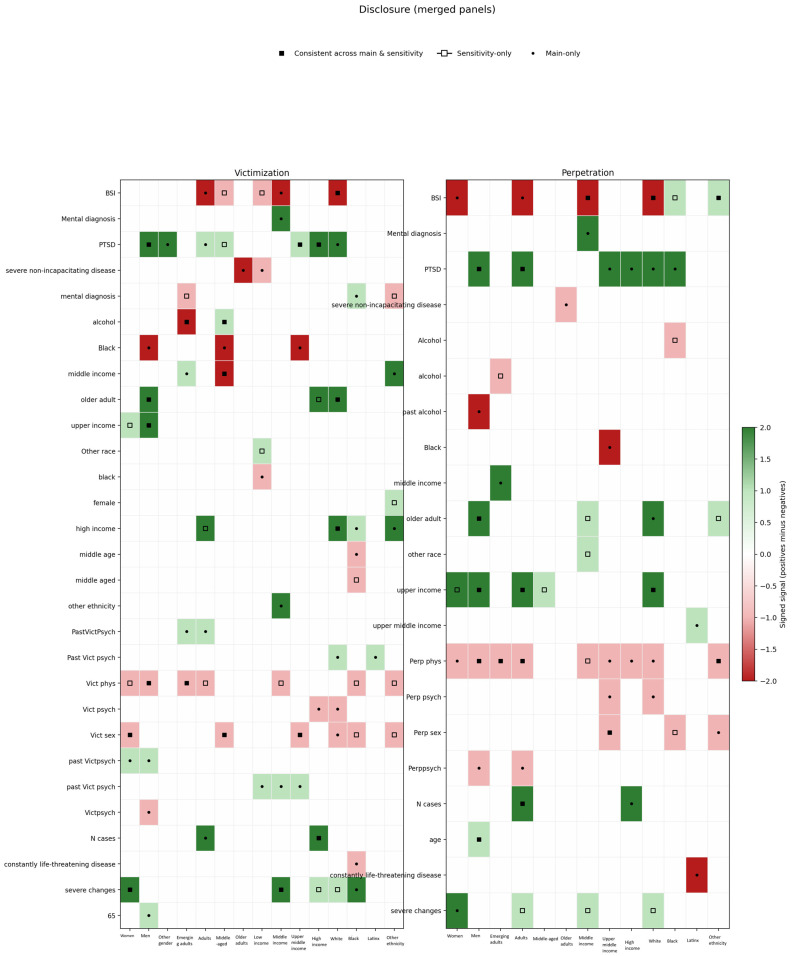
Robust correlates of disclosure intent by demographic group.

Only the gender minorities group (i.e., “other”) reported significantly lower disclosure intent when using the E-HITS. J-IPV showed a higher disclosure intent of women. Among males, PTSD, upper income and older age were robustly correlated with higher disclosure intent consistently. Only in the main analysis, psychological and physical violence (both perpetration and victimization), being a Black victim and a perpetrator with history of alcohol abuse were negatively correlated with disclosure intent. For females, severe changes in daily life had consistent positive association with disclosure. Physical and sexual victimization were consistently negatively correlated. Upper income emerged as a positive correlate only in the sensitivity analysis, and BSI had some negative association with the outcome among perpetrators. For gender minorities, there were too few participants to support all the models in the sensitivity analysis of consistent reporting, but in the main analysis PTSD was a robust positive correlate among victims. The significant correlates by gender are presented in Figure 2.

#### 3.3.3. RQ 2.3: Did Correlates of Disclosure Intention Differ by Age (Life-Stage)?

Emerging adults showed overall significantly lower disclosure intent. The J-IPV also showed a lower disclosure intent amongst adults compared to older adults. Older adult survivors were significantly different from adults and middle aged. In emerging adults, physical violence (both perpetration and victimization) was a robust negative correlate of disclosure; in the main analysis middle income was a consistent positive correlate, and in the sensitivity analysis of consistent answers alcohol abuse was a negative correlate. In adults, upper income and PTSD had a robust positive association, and physical perpetration a robust negative association with disclosure intent. In the main analysis number of cases in the state was a consistent positive correlate and BSI a consistent negative. Among the middle aged, there were no consistently significant correlates for perpetrators. For victims the most robust correlates were middle income, being Black and sexual victimization, all of which were negatively correlated with help-seeking. Among the older adults, severe non-incapacitating diseases were negative correlates of disclosure intent in the main analysis, especially among victims. The significant correlates by age are presented in Figure 2.

#### 3.3.4. RQ 2.4: Did Correlates of Disclosure Intention Differ by Income Level?

There were significant differences between income level groups; generally lower income groups had lower disclosure intent. In the low-income group, no correlates reached significance for perpetrators of any IPV type and victims of sexual IPV in any analysis. Severe non-incapacitating disease and being Black were negatively correlated with disclosure intent among victims of psychological IPV and past psychological victimization was positively correlated in the main analysis. BSI was a negative correlate and “other” ethnicity a positive correlate among victims of physical IPV in the sensitivity analysis. Among those with middle income, most correlates reached significance primarily in the main analysis. These were consistently BSI that was associated with lower disclosure intent, and mental diagnosis and severe changes in daily life, which were positively associated with disclosure intent. In the upper-middle-income group, sexual violence was a robust negative correlate in line with the full sample, and PTSD was a less consistent positive correlate. Being Black was also a negative correlate in the main sample. Among those with high income, PTSD and number of cases had a consistent positive association and were confirmed in some of the sensitivity analysis models, especially for victims. Older age had a positive association with intent in some analyses of victims. The significant correlates by income are presented in Figure 2.

#### 3.3.5. RQ 2.5: Did Correlates of Disclosure Intention Differ by Ethnicity?

When using the E-HITS in the main analysis, Black participant survivors and those of “other” ethnicity showed lower disclosure intent than the White and multi-ethnic participants. The same held for perpetrators, with the addition that Latinx perpetrators showed lower intent than the White and Native. These findings were not confirmed in the sensitivity analysis with the consistent cases. In terms of correlates, in the White participants group, BSI score had a robust negative correlation, as in the full sample, and upper income, older age and PTSD were robust positive correlates of disclosure. Most types of violence were negative correlates. Among Black perpetrators, PTSD was a consistent positive correlate in the main analysis only. Severe changes in daily life had some positive association with disclosure for victims. Sexual violence and physical victimization were negative correlates of the outcome in the sensitivity analysis. Middle age was a consistent negative correlate of the outcome among victims. Among the Latinx participants, in the main analysis life-threatening diseases had a robust negative correlation among perpetrators, but among survivors almost no variable reached significance in any analysis. In the main analysis of survivors of other ethnicities middle and high income were significant positive correlates, and less robust ones were sexual victimization and PTSD. Physical and sexual violence were robust negative correlates among perpetrators, but for victims they appeared only in the sensitivity analysis of consistent answers. The significant correlates by ethnicity are presented in Figure 2.

## 4. Discussion

The findings in summary showed that higher income (upper middle and high), number of cases in the state and having faced changes in family life or becoming unemployed due to the pandemic were the most robust correlates of both outcomes. PTSD symptoms were robustly positively linked with help-seeking intent, and with disclosure intent when considering all individuals with IPV experiences, even if they are not at a stage to report them consistently. Disclosure was associated negatively with physical and sexual violence. The number of cases in the state had a more positive impact on the help-seeking intent of primarily more privileged demographic groups such as males, people of White ethnicity, age 30–44, high income, and also on people with lower middle income for help-seeking intent, who are probably more susceptible to external circumstances than those with more resources. Inconsistencies in self-reported violence across different scales significantly impacted results. This issue is highlighted particularly among emerging adults, middle-aged, older, people of middle and upper-middle income, non-White participants, and perpetrators. The lower help-seeking and disclosure intent of women, gender minorities, Black individuals and emerging adults highlight their vulnerability and a need for additional attention.

All in all, external factors and resources, i.e., the number of COVID-19 cases in the state and higher income, impacted more help-seeking intent, especially in the analysis of all participants regardless of consistency in reporting. The highly considerable nuances across demographic groups illustrate that some, such as the older adults and those with middle income, are likely more affected by this external situation and that others, such as men and people of age 30–44, have the capacity to adapt to the external circumstances more. Changes in daily life are an in-between factor for the external and intra-individual effects, and they have a greater importance for victims, women, emerging adults, and those with middle income both for help-seeking and disclosure intent. This is definitely a non-surprising finding, as changes in daily life were widely considered a main driver of the different impacts of the pandemic. It is also intuitive that they would constitute an incentive to consider taking action when being victimized, given the additional pressure. The positive impact of number of COVID-19 cases on help-seeking was unexpected, and it diverges from the literature showing decreased contacts with healthcare services during the first and strict stages of the lockdown [58,107,108]. It is probably explained by the fact that this study measured intention, instead of actual behavior; most importantly, the phrasing of the questions “Were a healthcare provider to ask you such questions…” refers to a positive scenario where a healthcare provider offers resources, and it sounds as if the availability of these resources is not questioned, and this barrier could thus be considered as skipped. In such a case, a provider offering resources might come as a positive surprise, eagerly accepted. It may also be due to a sense of urgency that could have been created in states overburdened with COVID-19.

PTSD symptoms had mostly positive associations, which is in line with the previous findings of mental ill health as motivation to accessing care [45,109,110], and especially the high association of PTSD with service use [99,111]. Maybe the PTSD symptoms facilitate the recognition of mental-health-related struggle and thus contribute to help-seeking. The negative correlations of the BSI score are also in line with the literature and the theories that support that mental ill health can have a negative or varied impact on help-seeking [44,46]. The negative correlations of mental-health-related variables that could be interpreted as pointing to the fear and stigma that are frequently found in the literature as barriers to disclosure [24,25,112,113,114]. The seeming contradiction of the positive associations of PTSD symptoms and the negative associations of common mental distress symptoms as measured by the BSI has been explained articulately by Fleming and colleagues [49], who found that more avoidant symptoms, which are dominant in common manifestations of distress such as depressive and anxious symptoms, were negatively linked with help-seeking, while more “active” PTSD symptoms were positively linked with help-seeking. The finding that BSI score negatively correlated with disclosure intent in the main analysis with all participants that reported IPV, even inconsistently across scales, might point to some of the reasons for the inconsistent reporting; ill mental health may cause people to feel uncertainty about whether their experiences can be deemed as abusive. The nuances across demographic groups are also remarkable, as BSI score is more impactful among women, especially perpetrators, people of low and middle income, ages 30–44 and White ethnicity. For women and people of lower income it might be because of more common occurrence of mental ill health within these groups, while for the White individuals and those 30–44 it might be that there are fewer barriers to care overall, and avoidant symptoms are one of the main difficulties in accessing care. The positive correlations of having a mental diagnosis with disclosure for some groups might be an indication of familiarity with the health system and maybe increased access to care.

The results on negative impact of violence initially appear counterintuitive and incongruent with the evidence of severity of violence being highly correlated with disclosure and help-seeking [109,115,116] and also from some evidence of increased disclosure during the pandemic by survivors of coercive control [117]. However, the present study did not assess the severity of physical and sexual violence, or the exact concept of coercive control, so it remains unclear. It might also be due to the differences between intent and actual behavior. Another possible explanation given the high inconsistency in the reporting of violence, is that many of the participants in this study did not feel sure if their experiences were worth disclosing to and discussing with a healthcare provider. Alternatively, given that these data were collected at the earliest stage of the pandemic, people could still be feeling shocked and believing that they could not deal with IPV at the given moment, or they might have felt disempowered.

Physical health appears to be of less importance than expected in the context of the pandemic, but this can be attributed to the fact that demographic differences were controlled for even in the main models. Indeed, in the subgroup analyses of older adults, severe disease was consistently negatively correlated with disclosure, which is exactly what would be expected, given that older adults were the most vulnerable to COVID-19 and would prioritize their physical health. Having a life-threatening disease was negative correlate of disclosure among Latinx perpetrators and Black victims as well, which is possibly linked with unfavorable medical insurance, as a structural barrier to help-seeking [118], or prior negative experiences with healthcare providers [119]. Additionally, the fact that physical health was not correlated with the outcomes in the full sample could also be traced back to the phrasing of the questions as intent, rather than behavior, referring to a hypothetical scenario where they did have contact with a healthcare provider.

Another noteworthy finding was that the correlations were often similar for survivors and perpetrators, but this could be attributed to the high prevalence of bidirectional violence in the sample, that was inflated by the use of the CTS. This fact might also account for the underscored role of PTSD symptoms in this study, as PTSD is a very important factor in bidirectional violence [50].

An element that stands out is the considerable differences across sensitivity analyses, which highlights the fact that many people do not report on violence consistently across scales. This could be a sign of hesitation to disclose or uncertainty as to whether their experiences qualify as abuse. The phrasing of the questions appears to matter, which is not surprising given how challenging it can be to talk about abuse. This might mean that it would be even more difficult in a healthcare service, especially in the context of the pandemic, compared to the anonymity of an online survey. Emerging adults, Black participants and Latinx participants’ pronounced inconsistency is likely indicative of a vulnerability by being reluctant to disclose for a number of reasons, such as doubting if their experiences are abuse, or fear of legal consequences for perpetrators, or prior negative experiences with services [4,119,120]. Among female participants more inconsistency was observed for perpetration, which is probably because many of these behaviors were self-defense. The underlined inconsistency of the higher income groups is more pronounced among perpetrators, which is more intuitive, as they might not recognize that their behavior is abusive, or due to social desirability bias.

Regarding the overall demographic differences, higher income was one of the most robust positive correlates, underlining the overwhelming impact of inequalities, especially in the context of the United States, where healthcare is largely private and insurance bound. Men in this sample appear to be more empowered to disclose IPV victimization than described in the literature, where men have been found to face elevated stigma [98]. This may be because the pandemic and its socio-economic consequences had an overall lower burden on men [121]. The lower help-seeking and disclosure of women might be due to factors that were not thoroughly investigated in this study, such as having to care for children [122,123], or financial issues and dependence from their partner. These were reflected in the variable of changes in daily life that was one of the strongest positive correlates for both help-seeking and disclosure among women. Being Black was negatively correlated with both outcomes in many models, which is in line with the literature. Interestingly, even having upper-middle income did not seem to remove barriers for Black individuals, which highlights structural inequalities that are heightened in emergency contexts, far beyond mere financial inequalities. In addition, being middle-aged was very often negatively correlated with the outcomes in the subgroup analyses of Black ethnicity and vice versa. This illustrates the impact of intersectionality that merit further investigation. There were also fewer significant correlates of disclosure among the Black participants and the Latinx participants in the main analysis. That might mean that other factors, possibly immigration status or trust and access to the services, are more determining for these groups. It was also challenging to detect significant correlates for the low-income group, despite having sufficient participants, which could also reflect that factors not investigated in this study, such as financial dependency on the partner, which could be more determining [27,114].

People of age 30–44 also seem to be quite empowered and relatively more open to seeking help, and the same holds for the higher income groups, who appear less influenced by external factors and violence, and where mental health issues serve as motivation to open up to options. On the other end, emerging adults (18–29) seem to be less open to seeking help and disclosing, which corroborates the findings of some of the previous studies [97], but is at odds with others [17], presumably because it is a group more negatively affected by poor mental health, especially survivors. Older adults appear more prone to seeking help and disclosing, likely due to the increased sense of urgency, which was the highest for them. Overall, the results of the subgroup analyses point towards a sharpening of pre-existing economic and racial vulnerabilities and inequalities, which is congruent with qualitative research among Black participant survivors [124] and minorities in general in high-income countries [125,126].

These findings should be interpreted in the light of the strengths and limitations of this study. The major strength is that the individuals were reached directly, instead of inferring the barriers to help-seeking just by the service use records. However, the limitations are not to be overlooked. First and foremost, the phrasing of the outcome questions skips the reality of limited services and touch more upon an ideal scenario, and also intent does not always translate to behavior. That is due to the fact that the survey was designed prior to the outbreak of the pandemic and these questions were meant to investigate the acceptability of different phrasings. Secondly, the cross-sectional design allows only for associations and does not allow causal inference. Thirdly, the statistical analysis chosen is not fit for examining mechanisms and interconnections among variables. Another important source of doubt is that multiple models were tested, which increases the chance of Type I error and some variables reaching significance due to chance. For that reason we tried to report and emphasize mostly the results that were replicated across models. Lastly, the internet panel is essentially a convenience sample, and despite the quota sampling, the sample was found not to be exactly comparable to the USA population, so the findings might not be exactly generalizable. Older adults and people from very low- and very high-income margins are usually missed from such panels [127]. Furthermore, this is from the earliest stage of the pandemic, and it is known that the IPV and help-seeking patterns evolved throughout the duration of the pandemic, so it reflects only the early stages of emergency situations.

To overcome the pitfalls of this study, future research in potential future emergency situations should use longitudinal data when possible in order to establish the temporal order. Surveys should inquire about actual behavior as well, in order to examine to what extent intention translates into behavior. Since the COVID-19 pandemic has ended, it would be helpful to conduct research with records of civil society organizations and/or services on requests that were not fully met and contacts that were not followed, with a focus on demographic differences. Structural equation modeling would also be a useful statistical technique, as it would allow for investigating more complex relationships among the variables, but resource constraints did not allow for it in this study. Factors influencing help-seeking among women, different age groups, lower income groups, and people of color in the context of the pandemic warrant further investigation. Further research on intersectionality is also necessary, especially given that the results show clearly that pre-existing inequalities, especially those related to gender, age, income level and ethnicity, persist in the context of the pandemic, and qualitative studies provide evidence towards that direction [7]. Moreover, it was not possible to thoroughly investigate the help-seeking and disclosure intent for gender and sexual minorities, but there is an indication that their disclosure intent is lower, and that PTSD symptoms promote disclosure, so it would be important to investigate that further.

Despite these limitations, valuable practical recommendations remain. Most importantly, as recommended by a large number studies from the pandemic, IPV screening should be included in the academic courses that clinicians such as general practitioners take as part of their mandatory education [34]. That should be relatively feasible, as it is easier to integrate such elements in an academic course, rather than deliver additional training to clinicians with heavy workloads that are probably less inclined to adopt new practices. The phrasing also does matter considerably [92], as it is a key takeaway from our study too. We recommend the phrasing of the J-IPV tool [90], based on the findings of a recent scoping review [92]. Plus, it is important to screen in the presence of mental distress even if there are no signs of physical abuse [128], and also because it is already evident that provision of online services in the pandemic context ought to look out for survivors, instead of waiting for them to reach out [129]. There is an indication that people aged 18–29 and those of lower income face more barriers to help-seeking, and they should be prioritized. Emerging adults, the highest risk age group for IPV [130], should be prioritized. Men appear to be comparably involved in IPV, both as perpetrators and survivors, so providers should not neglect them, but instead abolish stereotypes towards them. Screening that builds upon trauma symptoms while remaining mindful of overall distress symptoms has a higher chance of being successful, as traumatic stress symptoms have a strong positive association with help-seeking and disclosure intent for many demographic groups, but overall distress and mental disorders have more negative correlations. It would also be helpful if mental health services focused more on screening for IPV, especially because mental health appears so closely intertwined with IPV. Given the rise in online mental health interventions, integrating IPV screening and service linkage is crucial, as survivors—especially vulnerable groups—report willingness to engage online [131,132]. Online screening seems more feasible in emergency contexts [133], but it has also been shown to be more acceptable and effective [134]. Also, telehealth consultations should include IPV screening with validated safety protocols. This should be implemented by taking serious privacy precautions, given that concerns on safety and privacy issues of online services have been raised in the context of the pandemic [14,135]. However, it is important that some in-person services continue flexibly through collaboration of different services and conscious proactivity of providers [136], not to heighten the digital inequalities [129]. In order to try to reach more vulnerable populations, community-based approaches for delivery and automated services for appointment scheduling can be implemented, which were feasible during the pandemic [137,138].

## 5. Conclusions

The context of the COVID-19 pandemic was a challenge for IPV victims/survivors and healthcare providers, but when seen as an incentive for change, there is potential. Training clinicians in IPV screening already in their education, using the phrasing of J-IPV, safe screening in telehealth appointments, community-based delivery and automated outreach systems for scheduling appointments are highly recommended in the event of emergency situations, such as natural disasters or potential future health crises. Even more attention should be paid to historically vulnerable groups, because the services and programs that are typically restricted in emergency contexts are those that serve these groups [139], and they are those that face the most barriers to services from the systemic to the individual level. Retrospective research on records of minimal contacts with different services from the pandemic or studies with repeated measurements and structural equation modeling would enrich the lessons that must be learnt to prepare for future crises.

## Figures and Tables

**Table 1:** Research questions and sub-questions.

Main Research Question	RQ 1.1: What Correlated with Intention to Seek Help for IPV?	RQ 2.1: What Correlated with Intention to Disclose IPV?
Differences in the main research question by demographic variable	Gender	
	Age (life stage)	
	Income level	
	Ethnicity	

**Table 2:** Descriptives of the sample N = 1346.

Variable	N (%)/M (SD) (Range)
Gender	
Male	674 (50.2%)
Female	635 (47.3%)
Agender	11 (0.7%)
Genderfluid	6 (0.4%)
Gender identity not represented	4 (0.3%)
Transgender	3 (0.2%)
Two-spirit	3 (0.2%)
Another gender	2 (0.1%)
Gender expansive	2 (0.1%)
Genderqueer	1 (0.1%)
Non-binary/non-conforming	1 (0.1%)
Sexual Orientation	
Heterosexual	1037 (79.3%)
Bisexual	93 (7.1%)
Asexual	71 (5.1%)
Homosexual	51 (3.9%)
Sexual orientation/identity not represented	30 (2.0%)
Pansexual	10 (0.7%)
Questioning/Not sure	9 (0.6%)
Demisexual	4 (0.3%)
Two-spirit	2 (0.1%)
Age	
Adults 30–44	474 (35.3%)
Middle aged 45–64	365 (27.2%)
Emerging adults 18–29	332 (24.7%)
Older adults 65+	172 (12.8%)
Ethnicity	
White/European American	823 (61.2%)
Latinx/Caribbean	187 (13.9%)
Black/African American	174 (12.9%)
Multiracial	88 (6.5%)
East and Southeast Asian	39 (2.9%)
South Asian	13 (1%)
Other	10 (0.7%)
Native	
American/Alaskan/Hawaiian	7 (0.5%)
Middle Eastern	4 (0.3%)
Relationship status	
In a monogamous partnership	810 (60.4%)
Single	382 (28.5%)
Separated or divorced	98 (7.3%)
In a non-monogamous partnership	30 (2.2%)
Widowed	22 (1.6%)
Employment status	
Employed	966 (73.3%)
Retired/Disabled	194 (14.7%)
Unemployed	157 (11.9%)
Education	
Bachelor’s or Associate degree	481 (35.8%)
Vocational-some college no degree	293 (21.8%)
Master’s/Professional degree-doctorate	291 (21.7%)
High School Diploma	213 (15.8%)
Incomplete High School	66 (4.9%)
Annual household income	
Middle 29,001–69,000	452 (34.2%)
Upper middle 69,001–150,000	439 (33.2%)
Low 0–29,000 dollars	279 (21.1%)
High 150,000+	152 (11.5%)
Religion	
Catholic	555 (44.3%)
Protestant	290 (23.1%)
Other Christian	130 (10.4%)
Atheist	94 (7.5%)
Agnostic	59 (4.7%)
Jewish	40 (3.2%)
Muslim	39 (3.1%)
Other	30 (2.4%)
Hindu	17 (1.4%)
USA born	
No	130 (9.7%)
State	
New York	173 (14.3%)
California	148 (12.3%)
Texas	103 (8.5%)
Florida	104 (8.6%)
Georgia	52 (4.3%)
Illinois	45 (3.7%)
Pennsylvania	50 (4.1%)
Ohio	32 (2.6%)
New Jersey	37 (3.1%)
Other	464 (38.4%)
Number of cases in the state 1 May 2020	
24,000+	784 (64.9%)
10,000–23,999	181 (15%)
5000–9999	146 (12.1%)
1–4999	97 (8%)
Changes in daily life	
Mild changes (work/social life)	384 (44.9%)
Severe changes (lost job/changed family life)	275 (32.2%)
Had COVID-19/someone close had	345 (25.6%)
Suspected COVID-19	
Sought help	241 (18%)
Did not seek help	30 (2.2%)
Alcohol abuse	
Past	117 (9.6%)
Current	54 (4.4%)
PTSD score	6.42 (6.62) (0–24)
BSI score	18.47 (18.20) (0–72)
Anxiety disorder	
Past	278 (22.7%)
Current	80 (6.5%)
Depression	
Past	330 (26.8%)
Current	95 (7.7%)
PTSD diagnosis	
Past	112 (9.1%)
Current	44 (3.6%)
Other mental illness	
Past	154 (12.6%)
Current	37 (3%)
Suicide attempt	
Past	106 (8.6%)
Recent (last 6 months)	35 (2.8%)
Any mental disorder diagnosis	
Past	390 (29.6%)
Current	184 (14%)
Physical health	
History of a serious disease	445 (37.7%)
Severe non-incapacitating disease	120 (10.2%)
Constantly life-threatening disease	107 (9.1%)
Mild disease or >5 symptoms	83 (7%)
Psychological perpetration	1.59 (1.06) (0–3)
Past psychological perpetration	0.8 (1.00) (0–3)
Physical perpetration	
Current	545 (40.5%)
Past	38 (2.8%)
Sexual perpetration	
Current	418 (31.1%)
Past	17 (1.2%)
Psychological victimization	1.66 (1.07) (0–3)
Past psychological victimization	0.82 (0.99) (0–3)
Physical victimization	
Current	576 (42.8%)
Past	44 (3.2%)
Sexual victimization	
Current	449 (33.4%)
Past	20 (1.4%)
Bidirectional violence	1214 (90.3%)
Help-seeking intent (perp.)	3.79 (1.22) (1–5)
Help-seeking intent (vict.)	3.74 (1.26) (1–5)
Disclosure intent (perp.)	4.06 (1.02) (1–5)
Disclosure intent (vict.)	4.04 (1.04) (1–5)
Acceptability of screening (perp.)	3.46 (0.79) (1–5)
Acceptability of screening (vict.)	3.44 (0.79) (1–5)

**Table 3:** Help-seeking intent.

Predictor (Standardized β Coefficients (95% CI)	Psychological		Physical		Sexual	
BIC	Vict. 3940.25	Perp. 3933.2	Vict. 3941.25	Perp. 3935.42	Vict. 3940.11	Perp. 3938.34
Age (reference 18–29 Emerging adults)						
30–45 Adults	0.044 (−0.104, 0.193)	0.152 (−0.003, 0.306)	0.044 (−0.104, 0.192)	0.165 (0.018, 0.312) *	0.046 (−0.101, 0.194) *	0.168 (0.020, 0.315) *
46–64 Middle aged	−0.082 (−0.240, 0.077)	−0.059 (−0.230, 0.112)	−0.085 (−0.244, 0.073)	−0.035 (−0.193, 0.123)	−0.080 (−0.238, 0.077)	−0.022 (−0.180, 0.135)
65+ Older adults	−0.085 (−0.295, 0.124)	−0.026 (−0.248, 0.197)	−0.096 (−0.301, 0.109)	0.001 (−0.205, 0.208)	−0.089 (−0.292, 0.114)	0.023 (−0.182, 0.228)
Gender (reference male)						
Female	−0.139 (−0.255, −0.022) *	−0.082 (−0.200, 0.035)	−0.134 (−0.249, −0.019) *	−0.090 (−0.204, 0.025)	−0.134 (−0.249, −0.020) *	−0.090 (−0.205, 0.024)
Other gender	−0.166 (−0.541, 0.209)	−0.181 (−0.550, 0.188)	−0.171 (−0.538, 0.196)	−0.190 (−0.551, 0.171)	−0.176 (−0.543, 0.191)	−0.197 (−0.559, 0.164)
Income level (reference low < $29,000						
Medium $29,001–$69,000	0.084 (−0.066, 0.233)	0.127 (−0.022, 0.276)	0.072 (−0.077, 0.220)	0.124 (−0.024, 0.272)	0.071 (−0.078, 0.220)	0.132 (−0.016, 0.279)
Upper medium $69,001–$150,000	0.129 (−0.031, 0.289)	0.228 (0.071, 0.386) *	0.123 (−0.037, 0.283)	0.221 (0.064, 0.377) *	0.123 (−0.037, 0.283)	0.225 (0.068, 0.381) *
High $150,000+	0.287 (0.061, 0.513) *	0.280 (0.065, 0.496) *	0.262 (0.052, 0.471) *	0.289 (0.079, 0.499) *	0.261 (0.052, 0.471) *	0.294 (0.084, 0.504) *
Ethnicity (reference White)						
Black or African American	−0.033 (−0.209, 0.142)	−0.013 (−0.185, 0.159)	−0.030 (−0.200, 0.140)	−0.027 (−0.196, 0.141)	−0.031 (−0.200, 0.139)	−0.028 (−0.197, 0.141)
Latinx and Caribbean	−0.054 (−0.231, 0.124)	−0.033 (−0.204, 0.139)	−0.043 (−0.213, 0.127)	−0.027 (−0.196, 0.142)	−0.039 (−0.210, 0.131)	−0.027 (−0.196, 0.142)
Native American-Alaskan-Hawaiian	0.832 (0.093, 1.571) *	0.732 (−0.009, 1.472)	0.824 (0.092, 1.556) *	0.733 (−0.001, 1.468)	0.812 (0.080, 1.544) *	0.744 (0.007, 1.481) *
East and Southeast Asian	−0.052 (−0.386, 0.282)	0.011 (−0.308, 0.330)	−0.077 (−0.400, 0.246)	0.008 (−0.310, 0.325)	−0.079 (−0.402, 0.243)	0.012 (−0.306, 0.330)
South Asian	−0.035 (−0.579, 0.509)	−0.355 (−0.900, 0.191)	−0.035 (−0.579, 0.509)	−0.309 (−0.849, 0.231)	−0.029 (−0.572, 0.514)	−0.300 (−0.841, 0.240)
Middle Eastern	−0.294 (−1.274, 0.686)	0.264 (−0.712, 1.240)	−0.324 (−1.299, 0.651)	0.237 (−0.734, 1.209)	−0.290 (−1.266, 0.686)	0.250 (−0.724, 1.223)
Multiracial	−0.132 (−0.359, 0.095)	−0.060 (−0.285, 0.164)	−0.141 (−0.364, 0.082)	−0.078 (−0.301, 0.144)	−0.141 (−0.364, 0.082)	−0.070 (−0.293, 0.152)
Other ethnicity	0.103 (−0.534, 0.741)	0.049 (−0.581, 0.679)	0.110 (−0.518, 0.738)	0.006 (−0.619, 0.632)	0.110 (−0.518, 0.738)	0.001 (−0.625, 0.628)
Physical health (reference < 5 symptoms)						
Mild disease or >5 symptoms	0.056 (−0.197, 0.308)	0.134 (−0.107, 0.375)	0.067 (−0.176, 0.309)	0.125 (−0.109, 0.360)	0.069 (−0.174, 0.311)	0.118 (−0.117, 0.353)
Severe yet not incapacitating disease	−0.101 (−0.308, 0.105)	−0.064 (−0.273, 0.146)	−0.109 (−0.318, 0.100)	−0.068 (−0.274, 0.137)	−0.096 (−0.306, 0.113)	−0.071 (−0.277, 0.136)
Constantly life-threatening disease	−0.217 (−0.482, 0.047)	−0.206 (−0.464, 0.052)	−0.224 (−0.489, 0.041)	−0.231 (−0.488, 0.026)	−0.203 (−0.473, 0.066)	−0.219 (−0.481, 0.044)
History of a serious disease	0.035 (−0.107, 0.178)	−0.056 (−0.192, 0.081)	0.037 (−0.100, 0.175)	−0.053 (−0.187, 0.082)	0.038 (−0.099, 0.176)	−0.053 (−0.188, 0.081)
Changes in daily life (reference no changes)						
Changes in work and social life	0.131 (−0.057, 0.319)	0.082 (−0.100, 0.265)	0.134 (−0.052, 0.320)	0.073 (−0.106, 0.253)	0.138 (−0.049, 0.324)	0.073 (−0.107, 0.254)
Lost job or changed family life	0.280 (0.088, 0.472) *	0.074 (−0.119, 0.268)	0.276 (0.084, 0.467) *	0.082 (−0.103, 0.267)	0.275 (0.084, 0.466) *	0.076 (−0.112, 0.264)
Alcohol current	0.001 (−0.325, 0.328)	−0.071 (−0.377, 0.234)	0.005 (−0.311, 0.321)	−0.095 (−0.404, 0.214)	0.015 (−0.302, 0.331)	−0.099 (−0.410, 0.211)
Alcohol past	−0.117 (−0.335, 0.101)	−0.087 (−0.304, 0.129)	−0.117 (−0.328, 0.094)	−0.107 (−0.315, 0.100)	−0.112 (−0.323, 0.099)	−0.113 (−0.321, 0.095)
Any mental diagnosis	−0.026 (−0.094, 0.043)	−0.030 (−0.098, 0.038)	−0.028 (−0.096, 0.040)	−0.032 (−0.098, 0.035)	−0.029 (−0.097, 0.039)	−0.033 (−0.100, 0.034)
BSI total	−0.024 (−0.106, 0.058)	−0.036 (−0.119, 0.046)	−0.032 (−0.112, 0.048)	−0.047 (−0.127, 0.033)	−0.024 (−0.105, 0.057)	−0.047 (−0.128, 0.034)
N cases	0.102 (0.041, 0.164) *	0.073 (0.009, 0.137) *	0.100 (0.040, 0.161) *	0.073 (0.013, 0.133) *	0.099 (0.039, 0.160) *	0.073 (0.013, 0.133) *
PTSD score	0.135 (0.055, 0.214) *	0.173 (0.094, 0.253) *	0.137 (0.058, 0.217) *	0.172 (0.094, 0.250) *	0.140 (0.061, 0.219) *	0.167 (0.089, 0.245) *
Past psychological	0.034 (−0.123, 0.191)	−0.080 (−0.225, 0.065)	-		-	
Violence	−0.074 (−0.173, 0.026)	−0.041 (−0.127, 0.046)	−0.070 (−0.182, 0.042)	−0.133 (−0.248, −0.018) *	−0.107 (−0.239, 0.024) *	−0.106 (−0.247, 0.034)

Significant values are marked with bold and an asterisk.

**Table 4:** Disclosure intent.

Predictor (Standardized β Coefficients (95% CI))	Psychological		Physical		Sexual	
BIC	Vict. 3931.32	Perp. 3951.59	Vict. 3964.22	Perp. 3956.59	Vict. 3956.83	Perp. 3957.36
Gender (reference male)						
Female	−0.048 (−0.168, 0.073)	−0.026 (−0.145, 0.093)	−0.026 (−0.144, 0.092)	−0.030 (−0.148, 0.088)	−0.030 (−0.148, 0.088)	−0.037 (−0.155, 0.081)
Other gender	0.090 (−0.292, 0.472)	0.075 (−0.287, 0.438)	0.079 (−0.286, 0.444)	0.086 (−0.278, 0.450)	0.072 (−0.293, 0.436)	0.078 (−0.286, 0.442)
Age (reference Emerging adults 18–29)						
30–45 Adults	0.037 (−0.118, 0.192)	0.032 (−0.119, 0.184)	0.045 (−0.105, 0.196)	0.044 (−0.106, 0.194)	0.049 (−0.101, 0.199)	0.048 (−0.102, 0.199)
46–64 Middle aged	0.033 (−0.128, 0.193)	0.006 (−0.161, 0.173)	0.043 (−0.117, 0.203)	0.026 (−0.134, 0.186)	0.048 (−0.111, 0.207)	0.039 (−0.120, 0.198)
65+ Older adults	0.217 (0.005, 0.429) *	0.148 (−0.072, 0.368)	0.199 (−0.012, 0.409)	0.174 (−0.036, 0.384)	0.204 (−0.004, 0.412)	0.197 (−0.012, 0.405)
Income (reference low < $29,000)						
Medium $29,001–$69,000	0.030 (−0.126, 0.186)	0.012 (−0.137, 0.161)	0.001 (−0.148, 0.151)	−0.005 (−0.154, 0.144)	−0.003 (−0.153, 0.146)	0.005 (−0.144, 0.154)
Upper medium $69,001–$150,000	0.105 (−0.053, 0.262)	0.110 (−0.047, 0.268)	0.094 (−0.063, 0.251)	0.091 (−0.066, 0.248)	0.092 (−0.065, 0.249)	0.096 (−0.060, 0.253)
High $150,000+	0.300 (0.067, 0.533) *	0.249 (0.033, 0.464) *	0.233 (0.023, 0.443) *	0.232 (0.022, 0.441) *	0.230 (0.020, 0.439) *	0.239 (0.029, 0.448) *
Ethnicity (reference White)						
Black or African American	−0.220 (−0.392, −0.048) *	−0.175 (−0.350, −0.000)	−0.202 (−0.374, −0.030) *	−0.196 (−0.367, −0.025) *	−0.200 (−0.371, −0.029) *	−0.191 (−0.362, −0.020) *
Latinx and Caribbean	−0.083 (−0.260, 0.094)	−0.047 (−0.218, 0.124)	−0.041 (−0.212, 0.131)	−0.038 (−0.209, 0.132)	−0.034 (−0.205, 0.137)	−0.039 (−0.209, 0.132)
Native American-Alaskan-Hawaiian	0.233 (−0.523, 0.989)	0.186 (−0.551, 0.924)	0.165 (−0.574, 0.903)	0.160 (−0.577, 0.896)	0.137 (−0.600, 0.873)	0.177 (−0.559, 0.913)
East and Southeast Asian	0.043 (−0.295, 0.380)	−0.042 (−0.364, 0.280)	−0.053 (−0.377, 0.271)	−0.066 (−0.388, 0.257)	−0.053 (−0.376, 0.269)	−0.056 (−0.378, 0.267)
South Asian	−0.002 (−0.557, 0.554)	−0.025 (−0.581, 0.532)	0.002 (−0.554, 0.558)	0.013 (−0.541, 0.566)	0.003 (−0.550, 0.557)	0.022 (−0.531, 0.576)
Middle Eastern	−0.110 (−1.096, 0.875)	−0.087 (−1.068, 0.895)	−0.186 (−1.170, 0.799)	−0.158 (−1.139, 0.823)	−0.111 (−1.093, 0.871)	−0.112 (−1.094, 0.871)
Multiracial	0.066 (−0.168, 0.299)	0.045 (−0.182, 0.273)	0.041 (−0.185, 0.267)	0.038 (−0.187, 0.263)	0.038 (−0.187, 0.263)	0.049 (−0.176, 0.274)
Other ethnicity	−0.506 (−1.172, 0.160)	−0.447 (−1.114, 0.220)	−0.487 (−1.149, 0.175)	−0.493 (−1.154, 0.167)	−0.485 (−1.145, 0.176)	−0.504 (−1.165, 0.157)
Physical health (reference < 5 symptoms)						
Mild disease or >5 symptoms	0.101 (−0.138, 0.340)	0.122 (−0.117, 0.361)	0.126 (−0.107, 0.359)	0.133 (−0.100, 0.366)	0.133 (−0.099, 0.365)	0.125 (−0.108, 0.358)
Severe yet not incapacitating disease	−0.084 (−0.300, 0.131)	−0.102 (−0.322, 0.119)	−0.114 (−0.329, 0.102)	−0.096 (−0.312, 0.119)	−0.081 (−0.297, 0.134)	−0.087 (−0.303, 0.129)
Constantly life-threatening disease	0.032 (−0.249, 0.314)	0.034 (−0.240, 0.308)	−0.012 (−0.281, 0.257)	0.013 (−0.255, 0.281)	0.036 (−0.236, 0.308)	0.057 (−0.217, 0.331)
History of a serious disease	0.034 (−0.113, 0.181)	0.030 (−0.114, 0.175)	0.034 (−0.106, 0.174)	0.041 (−0.099, 0.180)	0.039 (−0.100, 0.178)	0.046 (−0.094, 0.186)
Changes in daily life (reference no changes)						
Changes in work and social life	0.120 (−0.065, 0.305)	0.141 (−0.045, 0.326)	0.139 (−0.048, 0.326)	0.145 (−0.039, 0.330)	0.143 (−0.044, 0.330)	0.144 (−0.041, 0.329)
Lost job or changed family life	0.261 (0.088, 0.434) *	0.257 (0.072, 0.441) *	0.260 (0.078, 0.443) *	0.259 (0.078, 0.440) *	0.255 (0.072, 0.438) *	0.241 (0.059, 0.424) *
Alcohol past	−0.130 (−0.374, 0.114)	−0.072 (−0.296, 0.151)	−0.122 (−0.341, 0.097)	−0.107 (−0.326, 0.111)	−0.108 (−0.326, 0.111)	−0.110 (−0.329, 0.109)
Alcohol current	−0.026 (−0.361, 0.309)	0.005 (−0.289, 0.298)	−0.012 (−0.318, 0.295)	−0.012 (−0.318, 0.293)	0.012 (−0.294, 0.319)	−0.015 (−0.321, 0.290)
Any mental diagnosis	0.008 (−0.061, 0.077)	0.007 (−0.062, 0.075)	0.008 (−0.060, 0.075)	0.005 (−0.063, 0.072)	0.004 (−0.063, 0.072)	0.001 (−0.067, 0.069)
BSI total	−0.084 (−0.171, 0.003)	−0.077 (−0.160, 0.006)	−0.107 (−0.188, −0.025) *	−0.095 (−0.176, −0.013) *	−0.088 (−0.170, −0.005) *	−0.087 (−0.169, −0.004) *
N cases	0.056 (−0.006, 0.119)	0.051 (−0.011, 0.113)	0.048 (−0.012, 0.109)	0.050 (−0.010, 0.110)	0.047 (−0.013, 0.107)	0.052 (−0.008, 0.112)
PTSD score	0.117 (0.037, 0.197) *	0.120 (0.039, 0.201) *	0.116 (0.036, 0.195) *	0.124 (0.044, 0.203) *	0.126 (0.046, 0.205) *	0.124 (0.045, 0.203) *
Past violence	0.131 (−0.005, 0.266)	−0.049 (−0.185, 0.088)	-	-	-	
Violence	−0.205 (−0.299, −0.111) *	−0.118 (−0.200, −0.035) *	−0.104 (−0.218, 0.009)	−0.194 (−0.312, −0.077) *	−0.217 (−0.350, −0.084) *	−0.225 (−0.368, −0.082) *

Bold formatting and asterisks indicate significant values.

## Data Availability

Data supporting these findings are available upon request from M.D.

## Abbreviations

The following abbreviations are used in this manuscript:

IPV	Intimate Partner Violence
PTSD	Post-Traumatic Stress Disorder
BSI	Brief Symptom Inventory
SPSS	Statistical Package for the Social Sciences
E-HITS	Extended-Hurt/Insult/Threaten/Scream
JIPV	Jellinek Inventory of Partner Violence
CTS	Conflict Tactics Scale
